# The Financial Impact of Pregnancy and Childbirth Among Louisiana Medicaid Beneficiaries

**DOI:** 10.1111/1475-6773.70166

**Published:** 2026-09-17

**Authors:** Brigham Walker, Ethan Tsai, Andrew Anderson, Melissa Evans, Kevin Callison

**Affiliations:** ^1^ Department of Health Policy and Management Tulane University New Orleans Louisiana USA; ^2^ Department of Health Policy and Management Johns Hopkins University Baltimore Maryland USA; ^3^ Department of Social, Behavioral, and Population Science Tulane University New Orleans Louisiana USA; ^4^ The Murphy Institute Tulane University New Orleans Louisiana USA

**Keywords:** childbirth, credit, debt, Medicaid, pregnancy

## Abstract

**Objective:**

To measure the impact of pregnancy and childbirth on debt and personal credit scores by race, ethnicity, and geography.

**Study Setting and Design:**

We used Medicaid claims data, credit report data, and the Callaway Sant'Anna difference‐in‐differences method to compare relative changes in credit score, medical debt, and nonmedical debt between women moving through their pregnancies and women who weren't pregnant.

**Data Sources and Analytic Sample:**

Louisiana Medicaid claims data (January 2017 to December 2020).

**Principal Findings:**

While outcomes were not adversely impacted for non‐Hispanic White women, non‐Hispanic Black women experienced worsening financial measures following their pregnancies: credit scores decreased from Month 4 to 14 (4 points, 95% CI: −5.65 to −1.81, *p* < 0.01), medical debt increased from Month 8 to 12 ($41, 95% CI: $6–$75, *p* = 0.02), and nonmedical debt increased from Month 2 to 21 ($44, 95% CI: $15–$74, *p* < 0.01).

**Conclusions:**

Pregnancy and childbirth were associated with worsening financial outcomes among low‐income Medicaid‐enrolled mothers, particularly among non‐Hispanic Black women. These findings highlight the importance of broader economic supports to reduce financial disruption associated with pregnancy and childbirth among low‐income families and address disparities in maternal and child well‐being.

## Key Points

- What is known on this topic?
  ○ Income loss during the postpartum period, often due to unpaid leave, can exacerbate financial strain.
  ○ Despite these potential challenges, little is known about how childbirth influences credit scores and debt.
  ○ Plausibly causal differences by race, ethnicity, and geography are also less well documented.
- What this study found?
  ○ Financial outcomes through pregnancy and childbirth were not adversely impacted for non‐Hispanic White women.
  ○ However, non‐Hispanic Black women experienced increased medical debt and nonmedical debt and worsening credit measures during pregnancy and childbirth.
  ○ While elevated nonmedical debt persisted among non‐Hispanic Black mothers through the end of the observation window, credit scores and medical debt began to recover within about 1 year after childbirth.

## 1 Introduction

Financial instability following childbirth is a common yet under‐studied challenge for low‐income families [1, 2]. Medical expenses, income disruptions, and increased caregiving demands can compromise economic security [1, 3, 4, 5]. Although Medicaid covers approximately 40% of US births and reduces out‐of‐pocket costs for care [6], health insurance alone may be insufficient to mitigate the financial distress that follows childbirth [7, 8].

Pregnancy and childbirth are periods of heightened financial vulnerability [7, 9]. Income loss during the postpartum period can exacerbate financial strain [10]. This burden is especially acute for low‐income families, particularly Black families, who are overrepresented in low‐wage jobs that typically lack paid parental leave [10, 11]. Enrollment in public assistance programs designed to ease financial stress remains underutilized among eligible populations [9, 12].

Measures such as debt and credit score offer insights into the financial impact of childbirth. Medical debt has been associated with premature mortality [13], reduced access to mental health care [14], and food and housing insecurity [15]. Credit scores are sensitive to major life events like childbirth [16]. These financial indicators may reveal unmet needs and help to identify families at risk of cascading financial instability.

This study examines the impact of childbirth on debt and credit scores among low‐income individuals insured by Medicaid. By linking administrative and financial data, we aim to provide new insights at the intersection of reproductive health, financial stability, and inequality.

## 2 Methods

### 2.1 Data and Sample

This study used Louisiana Medicaid claims data from January 2017 through December 2020 to identify continuously enrolled pregnant women (the treatment group, identified using delivery‐related Current Procedural Terminology [CPT] codes) and non‐pregnant women under 50 years of age (the control group, without any pregnancy‐related diagnosis or procedure codes). Pregnant women were defined as those whose first observed pregnancy episode resulted in a full‐term live birth. Mothers with stillbirths were excluded (Supporting Information Tables A1–A3).

Each subject was observed for 31 months across three periods: 9 months before pregnancy (the index period), 9 months of pregnancy plus the month of expected childbirth, and 12 months after childbirth. For nonpregnant women, an index date was randomly assigned to approximate the pregnant group distribution (see Supporting Information Table A4). All treated subjects were pregnant prior to the COVID‐19 pandemic. These claims data were then linked to monthly individual‐level credit report data from a national credit reporting bureau. The outcome variables included: (1) credit score, (2) total unpaid medical debt, and (3) total unpaid nonmedical debt.

We excluded those with more than 5 months of missing financial records within any of the three periods. After exclusions, 13.3% (10,209 of 76,468 individuals) had a missing value, for whom we imputed financial measures using their own quarterly mean outcome value. We further excluded those who relocated outside of Louisiana, had cancer, heart attack, or stroke diagnoses, and whose total unpaid debt values were above the 99th percentile. The final sample included 9717 pregnant women and 66,751 nonpregnant women. This study was reviewed by the Institutional Review Board of the first author's institution.

We employed the event‐study version of the Callaway and Sant'Anna Difference‐in‐Differences (CSDID) model [17], which accommodates staggered treatment timing, to estimate dynamic average treatment effects relative to 9 months prior to childbirth. We estimated the group‐time average treatment effect on the treated (ATT) for individuals getting pregnant at time *g* and whose outcomes were observed at time *t*, defined as

$$\mathrm{ATT}\left(g,t\right)=E\left[Y_{\mathrm{it}}-Y_{i,g-1}|G_{i}=g\right]-E\left[Y_{\mathrm{it}}-Y_{i,g-1}|G_{i}=\mathrm{NT}\right]$$

where *Y*_*i*_ is the outcome at time t and *Y*_*i,g‐1*_ is the outcome in the pretreatment period. The treatment group is defined by *G*_*i*_ *= g* and the control group is the “never‐treated group” *G*_*i*_ = *NT*. We then implemented a doubly robust inverse probability weighting (DRIPW) estimator, aggregated to the group‐time monthly ATT estimates to obtain the event‐study treatment effects. Covariates included age, age squared, race and ethnicity, rural and urban residence. Standard errors were clustered on the individual. We conducted stratified analyses by race and ethnicity (Black, White, and Hispanic) and residence (urban vs. rural) and combined race–residence groups (urban Black, rural Black, urban White, and rural White).

To compare measures between the cohorts, we reported means and standardized mean differences (SMDs) for (1) outcomes across the entire sample and (2) demographics and covariates at baseline (i.e., the month prior to pregnancy). We also conducted multiple robustness and sensitivity analyses: (1) excluding pandemic‐era observations, (2) restricting to those aged 18–40, (3) focusing on complete cases without imputation, and (4) utilizing alternative random assignment indices for the control group.

## 3 Results

This study included 76,468 continuously enrolled Medicaid beneficiaries in Louisiana, consisting of 9717 pregnant women and 66,751 nonpregnant comparators. The average age of the pregnant cohort was 26.1 years, 51% were Black, 36% were White, and 10% were Hispanic. In contrast, the nonpregnant cohort had an average age of 32.2 years, 48% were Black, 39% were White, and 9% were Hispanic. During the 31‐month observation window, the pregnant cohort had an average credit score of 558; 66% had poor credit with average total unpaid medical debt of $758 and nonmedical debt of $460. For the nonpregnant cohort, the average credit score was 559, 67% had poor credit with average unpaid medical and nonmedical debts of $856 and $510, respectively (Supporting Information Table A5).

**FIGURE 1:**
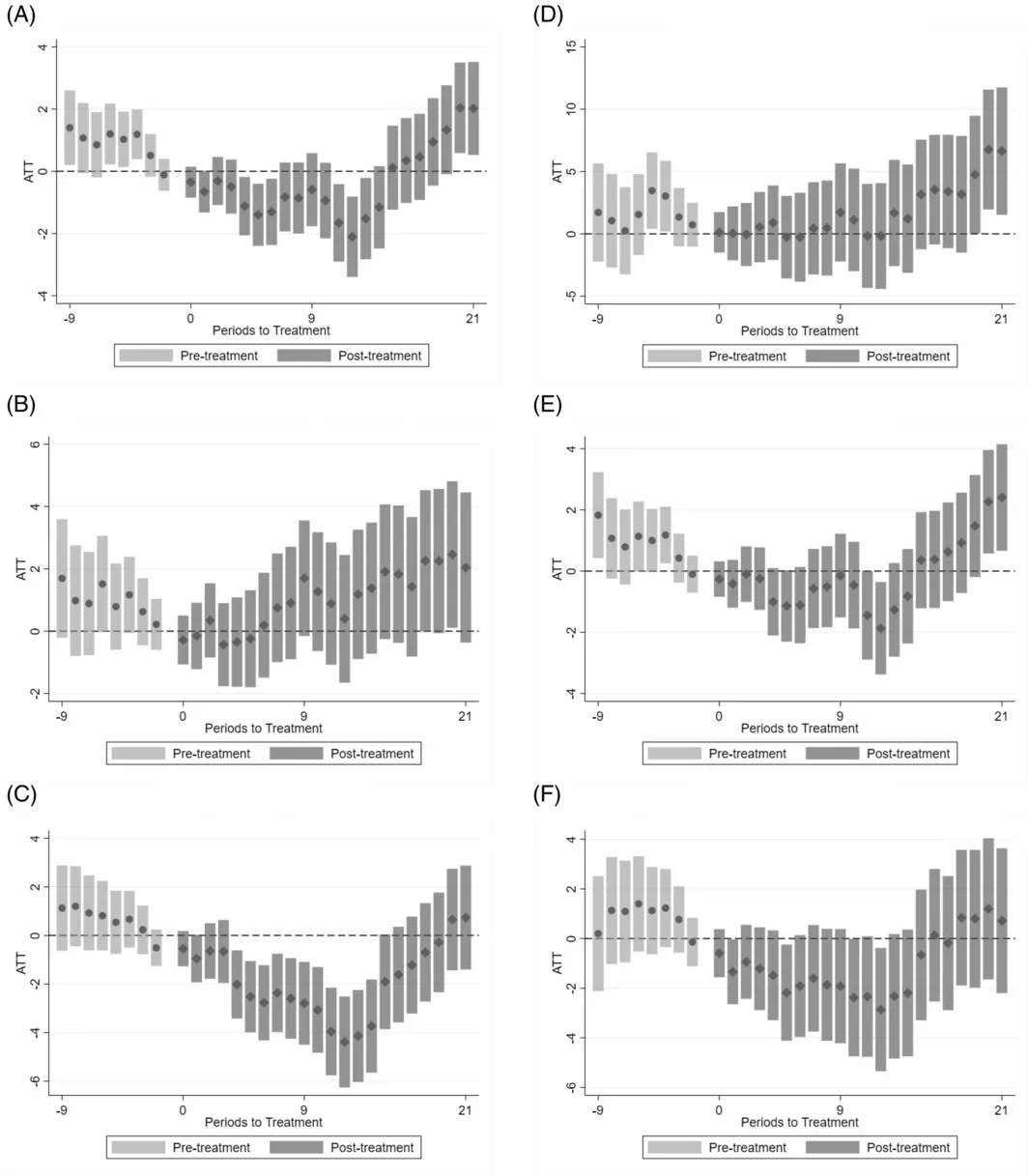
Credit score. (A) Overall. (B) White. (C) Black. (D) Hispanic. (E) Urban. (F) Rural.

Overall, pregnancy was associated with significantly lower credit scores during the pregnancy period, from Month 4 (−1.12, 95% CI: −2.06 to −0.18, *p* = 0.02) t0 6 (−1.31, 95% CI: −2.37 to −0.24, *p* = 0.02) and the post‐childbirth period, from Month 11 (−1.66, 95% CI: −2.90 to −0.41, *p* = 0.01) to 13 (−1.52, 95% CI: −2.83 to −0.21, *p* = 0.02). Credit scores overall improved during the post‐childbirth period, from Month 20 (2.04, 95% CI: 0.59–3.50, *p* = 0.01) to 21 (2.02, 95% CI: 0.53–3.52, *p* = 0.01) (Figure 1A and Supporting Information Table A6 [Column A]) driven by White and urban women (Figure 1B,E and Supporting Information Table A6 [Columns B and E]).

However, there were significant declines in credit scores among Black mothers from Month 4 (−2.02, 95% CI: −3.42 to −0.61, *p* = 0.01) to 14 (−3.73, 95% CI: −5.65 to −1.81, *p* < 0.01) (Figure 1C and Supporting Information Table A6 [Column C]). Additional analyses stratified by race and residence showed opposite patterns among urban women, with credit scores declining among Black mothers but increasing among White mothers (Supporting Information Figure A1). Although pregnant women had significantly higher credit scores than nonpregnant comparators in several pre‐period months, including Months −9 and −4 among urban women, neither the overall nor urban‐specific joint pre‐trend test rejected the null of no differential pre‐trends (*p* = 0.19 and *p* = 0.22, respectively).

**FIGURE 2:**
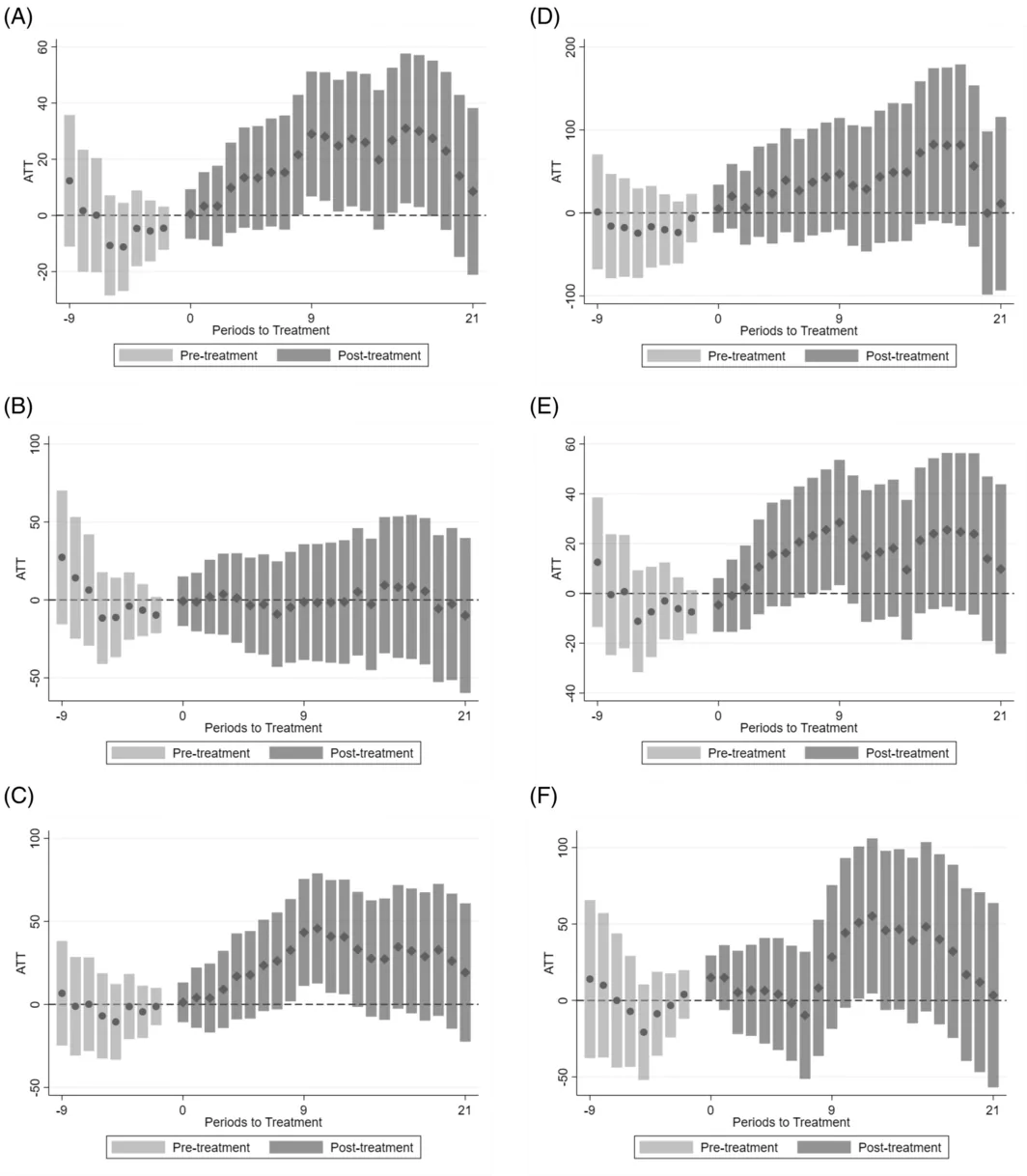
Medical debt. (A) Overall. (B) White. (C) Black. (D) Hispanic. (E) Urban. (F) Rural.

Medical debt levels increased by an average of about $29 during the post‐childbirth period, beginning in Month 9 ($29, 95% CI: $7–$51, *p* = 0.01) and remained elevated for most of the 9 months through Month 17 ($30, 95% CI: $3–$57, *p* = 0.03) (Figure 2A and Supporting Information Table A7 [Column A]). These increases were primarily observed among Black mothers, with average increases of about $40 between Month 8 ($33, $2–$64, *p* = 0.04) and 12 ($41, 95% CI: $6–$75, *p* = 0.02) (Figure 2C and Supporting Information Table A7 [Column C]). No such increases were observed among the White cohorts. There were instances of increased medical debt roughly coinciding with birth in both urban and rural cohorts (Figure 2E,F and Supporting Information Table A7 [Columns E and F]). The increase among urban women was primarily observed among Black mothers, while medical debt remained relatively stable among urban White mothers (Supporting Information Figure A2).

**FIGURE 3:**
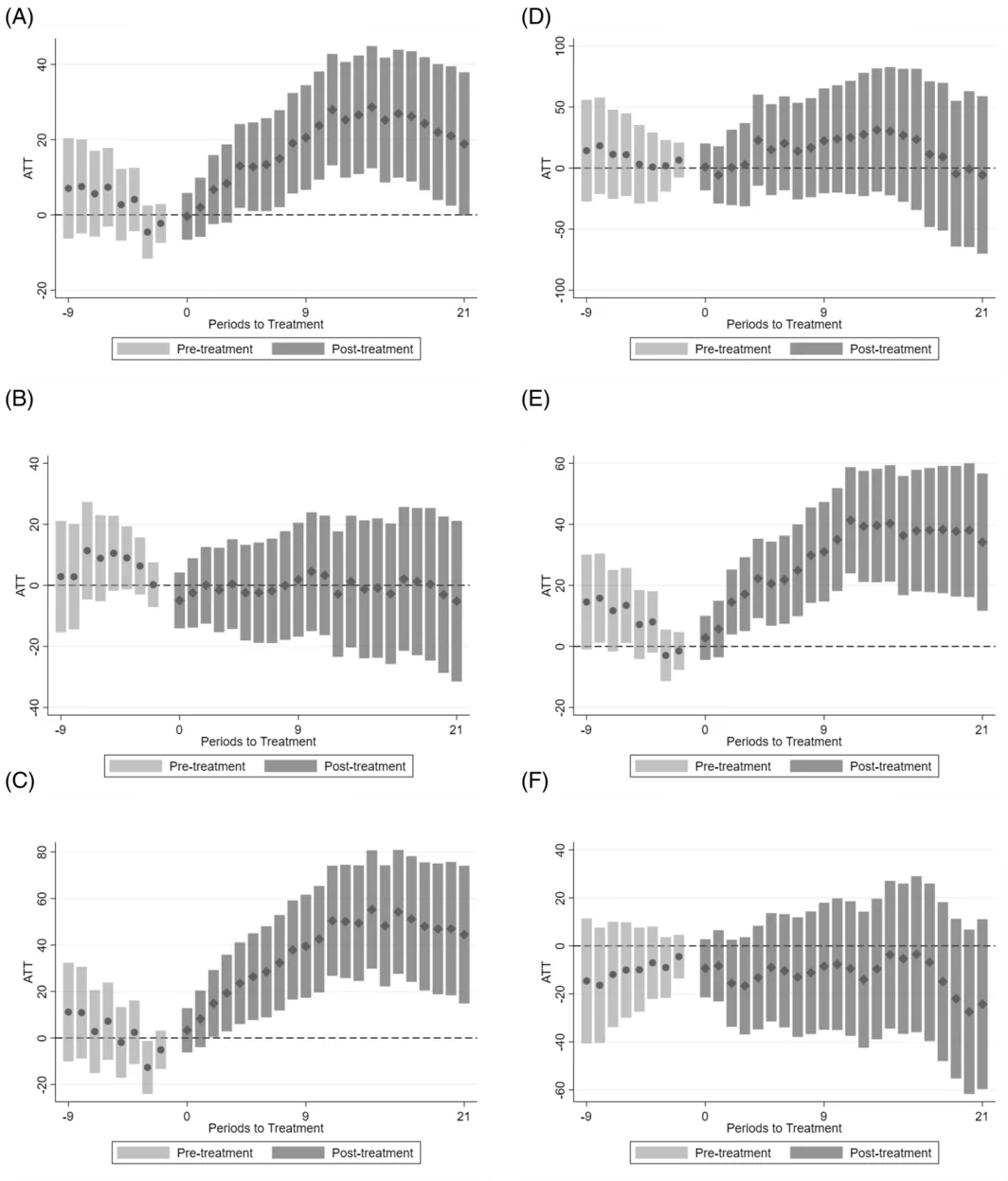
Nonmedical debt. (A) Overall. (B) White. (C) Black. (D) Hispanic. (E) Urban. (F) Rural.

Nonmedical debt levels increased by an average of about $13 during the pregnancy period, beginning in Month 4 ($13, 95% CI: $2–$24, *p* = 0.02) and persisted into the post‐childbirth period through Month 20 ($21, 95% CI: $2–$40, *p* = 0.03) (Figure 3A and Supporting Information Table A8 [Column A]). These increases were primarily observed among Black mothers, with average increases of about $15 from Month 2 (95% CI: $0.4–$29, *p* = 0.04) through the end of the study period in Month 21 ($44, 95% CI: $15–$74, *p* < 0.01) (Figure 3C and Supporting Information Table A8 [Column C]). White mothers did not experience any changes in nonmedical debt levels. Joint pre‐trend tests for nonmedical debt did not reject the null for Black or White women, although the *p* values were relatively close to the 0.05 threshold (*p* = 0.07 and *p* = 0.10, respectively).

Unlike medical debt, the nonmedical debt level grew among the urban population starting in Month 2 during the pregnancy period ($15, 95% CI: $4–$25, *p* = 0.01), climbing to about $40 in Month 14 (95% CI: $21–$59, *p* < 0.01) and persisting through the study period (Figure 3E and Supporting Information Table A8 [Column E]) while exhibiting no such patterns among the rural population. The increase among urban women was primarily observed among Black mothers (Supporting Information Figure A3). Pregnant urban women had significantly higher nonmedical debt than nonpregnant comparators in Months −8 and −6; however, the joint pre‐trend test was not statistically significant (*p* = 0.41).

For Hispanic women, joint pre‐trend tests rejected the null of no differential pre‐trends for credit score, medical debt, and nonmedical debt (all *p* < 0.01).

Results were substantively similar across all robustness and sensitivity analyses, including exclusion of pandemic‐era observations (Supporting Information Figures A4–A6), analyses among women aged 18–40 (Supporting Information Figures A7–A9), complete‐case analyses without imputation (Supporting Information Figures A10–A12), and an alternative random assignment of control‐group index dates (Supporting Information Figure A13).

## 4 Discussion

In this study of Medicaid beneficiaries, childbirth was associated with heterogeneous effects by race and ethnicity and, to a lesser extent, by geography. Non‐Hispanic White pregnant beneficiaries experienced no changes in medical or nonmedical debt and, if anything, saw slight increases in their credit scores. In contrast, non‐Hispanic Black mothers saw increases in both medical and nonmedical debt coincident with worsening credit scores.

Credit score declines among Black mothers were small against a cohort average of 558; however, where 66% already had poor credit (defined as below 580), small movements at this margin may have outsized impacts. The debt effects were proportionally larger—approximately 5% of the pregnant cohort's baseline medical debt and 10% of its nonmedical debt—and the nonmedical increase persisted through the end of the observation window. The findings for Hispanic mothers were not valid due to nonparallel pre‐trends. Urban mothers saw increases in nonmedical debt while rural mothers did not, and increases in medical and nonmedical debt among urban women were primarily observed among Black mothers.

While Medicaid coverage should substantially reduce direct medical costs, this study still observed an increase in medical debt among pregnant women. We cannot determine from these data why this has occurred, but it may be due to billing or administrative issues, care received from providers who do not accept Medicaid, or services not fully covered or successfully adjudicated. Further research is needed to distinguish these mechanisms directly.

These findings are consistent with national trends showing a rise in poverty from pregnancy to postpartum, especially among Black women, who are more likely to enter pregnancy in poverty, earn less, work part‐time jobs unlikely to offer paid maternity leave, and experience higher rates of pregnancy‐related morbidity [18, 19, 20, 21]. These structural disadvantages can increase the risk of poverty and job loss around the time of childbirth [9, 22]. The observed increase in debt and decline in credit scores among Black mothers may reflect the greater financial strain of childbirth as a disruptive event, particularly for families with fewer economic buffers and more severe health‐related work disruptions.

Louisiana does not mandate statewide paid leave wage replacement for pregnant or postpartum workers, leaving paid leave access largely dependent on employer‐provided benefits. Among women who worked during pregnancy in Louisiana, 48% used unpaid leave only after childbirth, 15% reported that they could not financially afford to take leave, and 35% reported that their job did not offer paid leave. These patterns support income disruption around childbirth as a plausible mechanism for increased debt; however, this study cannot directly observe employment status or maternity leave access in our data [23].

Participation in government supported programs like the Special Supplemental Nutrition Program for Women, Infants, and Children (WIC), Supplemental Nutrition Assistance Program (SNAP), and Temporary Assistance for Needy Families (TANF) may help to mitigate these financial effects following childbirth [9, 24]. However, barriers such as administrative complexity, stigma, and lack of awareness contribute to low uptake among eligible populations [12], and benefit levels are modest and unlikely to fully offset lost income, especially for families with low earnings before birth. Even with support from social programs, postpartum Black mothers may still experience higher poverty rates than postpartum White mothers [9].

The findings suggest that policies aimed at stabilizing income and simplifying access to existing support programs may help reduce the financial toll of childbirth. Public programs can align financial eligibility rules and adopt automatic enrollment processes to reduce barriers to access (e.g., women start receiving WIC benefits as soon as they have a Medicaid‐covered birth). Implementing guaranteed paid family leave would also allow low‐income families the opportunity to care for and bond with their newborn while preventing income shocks that contribute to credit score declines and rising debt [25].

### 4.1 Limitations

This study has several limitations. First, to avoid potential confounding driven by churn, we focused on women who were continuously enrolled, which may limit external validity. Our focus on Louisiana Medicaid enrollees without cancer, heart attack, stroke, and without extreme debt values similarly restricts broader generalizability. Second, the study also used 9 months to standardize the pregnancy period, but ultimately this merely approximates actual pregnancy durations. Third, several variables were not available in our dataset, including gestational age codes (Z3A.xx), pregnancy‐related conditions, household size, and so forth. Fourth, the credit data do not capture informal borrowing or unreported missed bills. Fifth, despite using a quasi‐experimental design, unobserved differences between cohorts may still confound the results. Sixth, we imputed a small share of missing financial data using quarterly means which risks smoothing over meaningful variation. Seventh, we do not report overlap diagnostics for the estimated propensity scores because the Callaway–Sant'Anna estimator estimates propensity scores separately across multiple group–time comparisons. However, replicating the analysis among women aged 18–40 (Supporting Information Figures A7–A9) addressed the largest observed covariate imbalance in age (SMD = 0.87), and the results were substantively unchanged. Eighth, because multiple outcomes, time periods, and subgroups were examined, some individually significant estimates may reflect chance. Lastly, we cannot directly observe access to or uptake of public benefits like WIC or SNAP, limiting our ability to assess how these programs mediate financial trajectories.

## 5 Conclusion

Pregnancy and childbirth for Medicaid‐enrolled families disproportionately financially impact non‐Hispanic Black mothers. While some financial outcomes recover within a year, the unequal burden observed across racial and geographic lines points to underlying differences in vulnerability and support. Strengthening public support systems through automatic enrollment, income stabilization, and paid leave may help reduce the financial disruption of childbirth.

## Funding

This work was supported by Commonwealth Fund, #24‐24156.

## Ethics Statement

This research has been reviewed by the Tulane and Louisiana Department of Health IRBs.

## Conflicts of Interest

The authors declare no conflicts of interest.

## Supporting information


**Table A1:** CPT codes for live birth delivery.
**Table A2:** ICD‐10‐CM codes for live birth and full‐term delivery.
**Table A3:** ICD‐10‐CM codes for stillbirth.
**Table A4:** Distribution of index year‐month by pregnancy status.
**Table A5:** Summary statistics.
**Table A6:** Impact of pregnancy on credit score.
**Table A7:** Impact of pregnancy on unpaid medical debt.
**Table A8:** Impact of pregnancy on unpaid nonmedical debt.
**Figure A1:** Credit score, by race and residence.
**Figure A2:** Medical debt, by race and residence.
**Figure A3:** Nonmedical debt, by race and residence.
**Figure A4:** Credit score, excluding the COVID‐19 pandemic period.
**Figure A5:** Medical debt, excluding the COVID‐19 pandemic period.
**Figure A6:** Nonmedical debt, excluding the COVID‐19 pandemic period.
**Figure A7:** Credit score among women aged 18–40.
**Figure A8:** Medical debt among women aged 18–40.
**Figure A9:** Nonmedical debt among women aged 18–40.
**Figure A10:** Credit score, complete cases without imputation.
**Figure A11:** Medical debt, complete cases without imputation.
**Figure A12:** Nonmedical debt, complete cases without imputation.
**Figure A13:** Alternative random assignment of control‐group index dates.

## Data Availability

Data are not publicly sharable.
