# Supplementary material for: The Financial Impact of Pregnancy and Childbirth Among Louisiana Medicaid Beneficiaries
Source: Health Serv Res. 2026 Sep 17;61(5):e70166. doi: 10.1111/1475-6773.70166 (PMC13583919; doi:10.1111/1475-6773.70166)
Supplement: Supplementary file 1 [file HESR-61-e70166-s001.docx]

**Appendix**

*Formal Empirical Specifications*

The DRIPW is defined as:

$$\text{AT}\text{T}_{\text{dr}}^{\text{NT}}\left( g,t \right)=E\left[ \left( \frac{G_{g}}{E\left[ G_{g} \right]}-\frac{p_{g}\left( X \right)\cdot C}{1-p_{g}\left( X \right)} / E\left[ \frac{p_{g}\left( X \right)\cdot C}{1-p_{g}\left( X \right)} \right] \right)\cdot\left( Y_{t}-Y_{g-1}-m_{g,t}^{NT}\left( X \right) \right) \right]$$

where *C* is an indicator for whether individual *i* belongs to the never-treated group (control), and *G_g_* is an indicator for being in group *g*. Individual fixed effects difference out time-invariant personal characteristics. *p_g_(X)* denotes the estimated propensity score*.* The term $m_{g,t}^{NT}\left( X \right)$ is the outcome regression function by control group, defined as:

$$m_{g,t}^{NT}\left( X \right)=E\left[ Y_{t}-Y_{g-1} \mid X,C=1 \right]$$

We aggregated the group-time ATT estimates by event time *e=t-g* to obtain the event-study treatment effects:

$$\text{AT}\text{T}_{\text{e}}^{\text{event}}=\frac{\sum_{g,t:t-g=e} w_{g,t}\cdot\text{ATT}\left( g,t \right)}{\sum_{g,t:t-g=e} w_{g,t}}$$

where *w_g,t_* is a weight based on the number of treated observations used to estimate the group-time ATT(*g,t*).

| *Table A1: CPT codes for live birth delivery* | |
| --- | --- |
| 1960 | Anesthesia for vaginal delivery only |
| 1961 | Anesthesia for cesarean delivery only |
| 1962 | Anesthesia for urgent hysterectomy following delivery |
| 1963 | Anesthesia for cesarean hysterectomy without any labor analgesia/anesthesia care |
| 1967 | Neuraxial labor analgesia/anesthesia for planned vaginal delivery (this includes any repeat subarachnoid needle placement and drug injection and/or any necessary replacement of an epidural catheter during labor) |
| 1968 | Anesthesia for cesarean delivery following neuraxial labor analgesia/anesthesia (list separately in addition to code for primary procedure performed) |
| 1969 | Anesthesia for cesarean hysterectomy following neuraxial labor analgesia/anesthesia (list separately in addition to code for primary procedure performed) |
| 59050 | Fetal monitoring during labor by consulting physician (ie, non-attending physician) with written report; supervision and interpretation |
| 59051 | Fetal monitoring during labor by consulting physician (ie, non-attending physician) with written report; interpretation only |
| 59400 | Routine obstetric care including antepartum care, vaginal delivery (with or without episiotomy, and/or forceps) and postpartum care |
| 59409 | Vaginal delivery only (with or without episiotomy and/or forceps) |
| 59410 | Vaginal delivery only (with or without episiotomy and/or forceps); including postpartum care |
| 59412 | External cephalic version, with or without tocolysis |
| 59414 | Delivery of placenta (separate procedure) |
| 59430 | Postpartum care only (separate procedure) |
| 59510 | Routine obstetric care including antepartum care, cesarean delivery, and postpartum care |
| 59514 | Cesarean delivery only |
| 59515 | Cesarean delivery only; including postpartum care |
| 59618 | Routine obstetric care including antepartum care, cesarean delivery, and postpartum care, following attempted vaginal delivery after previous cesarean delivery |
| 59620 | Cesarean delivery only, following attempted vaginal delivery after previous cesarean delivery |
| 59622 | Cesarean delivery only, following attempted vaginal delivery after previous cesarean delivery; including postpartum care |
| 99436 | Attendance at delivery and first stabilization of newborn |
| 99440 | Newborn resuscitation |

| *Table A2: ICD-10-CM codes for live birth and full-term delivery* | |
| --- | --- |
| **Live birth** | |
| Z37.0 | Single live birth |
| Z37.2 | Twins, both liveborn |
| Z37.50 | Multiple births, unspecified, all liveborn |
| Z37.51 | Triplets, all liveborn |
| Z37.52 | Quadruplets, all liveborn |
| Z37.53 | Quintuplets, all liveborn |
| Z37.54 | Sextuplets, all liveborn |
| Z37.59 | Other multiple births, all liveborn |
| Z38.00 | Single liveborn infant, delivered vaginally |
| Z38.01 | Single liveborn infant, delivered by cesarean |
| Z38.1 | Single liveborn infant, born outside hospital |
| Z38.2 | Single liveborn infant, unspecified as to place of birth |
| Z38.30 | Twin liveborn infant, delivered vaginally |
| Z38.31 | Twin liveborn infant, delivered by cesarean |
| Z38.4 | Twin liveborn infant, born outside hospital |
| Z38.5 | Twin liveborn infant, unspecified as to place of birth |
| Z38.61 | Triplet liveborn infant, delivered vaginally |
| Z38.62 | Triplet liveborn infant, delivered by cesarean |
| Z38.63 | Quadruplet liveborn infant, delivered vaginally |
| Z38.64 | Quadruplet liveborn infant, delivered by cesarean |
| Z38.65 | Quintuplet liveborn infant, delivered vaginally |
| Z38.66 | Quintuplet liveborn infant, delivered by cesarean |
| Z38.68 | Other multiple liveborn infant, delivered vaginally |
| Z38.69 | Other multiple liveborn infant, delivered by cesarean |
| Z38.7 | Other multiple liveborn infant, born outside hospital |
| Z38.8 | Other multiple liveborn infant, unsp as to place of birth |
| **Full-term delivery** | |
| O60.20X0 | Term delivery with preterm labor, unspecified trimester |
| O60.20X1 | Term delivery with preterm labor, unspecified trimester |
| O60.20X2 | Term delivery with preterm labor, unspecified trimester |
| O60.20X3 | Term delivery with preterm labor, unspecified trimester |
| O60.20X4 | Term delivery with preterm labor, unspecified trimester |
| O60.20X5 | Term delivery with preterm labor, unspecified trimester |
| O60.20X9 | Term delivery with preterm labor, unspecified trimester |
| O60.22X0 | Term delivery with preterm labor, second trimester |
| O60.22X1 | Term delivery with preterm labor, second trimester |
| O60.22X2 | Term delivery with preterm labor, second trimester |
| O60.22X3 | Term delivery with preterm labor, second trimester |
| O60.22X4 | Term delivery with preterm labor, second trimester |
| O60.22X5 | Term delivery with preterm labor, second trimester |
| O60.22X9 | Term delivery with preterm labor, second trimester |
| O60.23X0 | Term delivery with preterm labor, third trimester |
| O60.23X1 | Term delivery with preterm labor, third trimester |
| O60.23X2 | Term delivery with preterm labor, third trimester |
| O60.23X3 | Term delivery with preterm labor, third trimester |
| O60.23X4 | Term delivery with preterm labor, third trimester |
| O60.23X5 | Term delivery with preterm labor, third trimester |
| O60.23X9 | Term delivery with preterm labor, third trimester |
| O42.02 | Full-term premature rupture of membranes, onset of labor within 24 hours |
| O42.92 | Full-term premature rupture of membranes, unspecified time |
| O47.1 | False labor at or after 37 completed weeks of gestation |
| O80 | Encounter for full-term uncomplicated delivery |
| *Table A3: ICD-10-CM codes for stillbirth* | |
| Z37.3 | Twins, one liveborn and one stillborn |
| Z37.60 | Multiple births, unspecified, some liveborn |
| Z37.61 | Triplets, some liveborn |
| Z37.62 | Quadruplets, some liveborn |
| Z37.63 | Quintuplets, some liveborn |
| Z37.64 | Sextuplets, some liveborn |
| Z37.69 | Other multiple births, some liveborn |
| Z37.1 | Single stillbirth |
| Z37.4 | Twins, both stillborn |
| Z37.7 | Other multiple births, all stillborn |
| O31.00X0 | Papyraceous fetus, unsp trimester, not applicable or unsp |
| O31.00X1 | Papyraceous fetus, unspecified trimester, fetus 1 |
| O31.00X2 | Papyraceous fetus, unspecified trimester, fetus 2 |
| O31.00X3 | Papyraceous fetus, unspecified trimester, fetus 3 |
| O31.00X4 | Papyraceous fetus, unspecified trimester, fetus 4 |
| O31.00X5 | Papyraceous fetus, unspecified trimester, fetus 5 |
| O31.00X9 | Papyraceous fetus, unspecified trimester, other fetus |
| O31.02X0 | Papyraceous fetus, second trimester, not applicable or unsp |
| O31.02X1 | Papyraceous fetus, second trimester, fetus 1 |
| O31.02X2 | Papyraceous fetus, second trimester, fetus 2 |
| O31.02X3 | Papyraceous fetus, second trimester, fetus 3 |
| O31.02X4 | Papyraceous fetus, second trimester, fetus 4 |
| O31.02X5 | Papyraceous fetus, second trimester, fetus 5 |
| O31.02X9 | Papyraceous fetus, second trimester, other fetus |
| O31.03X0 | Papyraceous fetus, third trimester, not applicable or unsp |
| O31.03X1 | Papyraceous fetus, third trimester, fetus 1 |
| O31.03X2 | Papyraceous fetus, third trimester, fetus 2 |
| O31.03X3 | Papyraceous fetus, third trimester, fetus 3 |
| O31.03X4 | Papyraceous fetus, third trimester, fetus 4 |
| O31.03X5 | Papyraceous fetus, third trimester, fetus 5 |
| O31.03X9 | Papyraceous fetus, third trimester, other fetus |
| P95 | Stillbirth |
| O36.4XX0 | Maternal care for intrauterine death, not applicable or unspecified |
| O36.4XX1 | Maternal care for intrauterine death, fetus 1 |
| O36.4XX2 | Maternal care for intrauterine death, fetus 2 |
| O36.4XX3 | Maternal care for intrauterine death, fetus 3 |
| O36.4XX4 | Maternal care for intrauterine death, fetus 4 |
| O36.4XX5 | Maternal care for intrauterine death, fetus 5 |
| O36.4XX9 | Maternal care for intrauterine death, other fetus |

| *Table A4: Distribution of Index Year-Month by Pregnancy Status* | | | | | | | | |
| --- | --- | --- | --- | --- | --- | --- | --- | --- |
| Year month | Pregnancy | |  | No Pregnancy | |  | No Pregnancy (reassigned) | |
|  | N | % |  | N | % |  | N | % |
| 201710 | 622 | 6.40 |  | 3,692 | 5.53 |  | 3,847 | 5.76 |
| 201711 | 692 | 7.12 |  | 3,602 | 5.40 |  | 3,719 | 5.57 |
| 201712 | 581 | 5.98 |  | 3,727 | 5.58 |  | 3,665 | 5.49 |
| 201801 | 570 | 5.87 |  | 3,810 | 5.71 |  | 3,725 | 5.58 |
| 201802 | 596 | 6.13 |  | 3,776 | 5.66 |  | 3,680 | 5.51 |
| 201803 | 570 | 5.87 |  | 3,756 | 5.63 |  | 3,639 | 5.45 |
| 201804 | 606 | 6.24 |  | 3,701 | 5.54 |  | 3,711 | 5.56 |
| 201805 | 494 | 5.08 |  | 3,646 | 5.46 |  | 3,630 | 5.44 |
| 201806 | 462 | 4.75 |  | 3,671 | 5.50 |  | 3,701 | 5.54 |
| 201807 | 474 | 4.88 |  | 3,678 | 5.51 |  | 3,680 | 5.51 |
| 201808 | 490 | 5.04 |  | 3,777 | 5.66 |  | 3,738 | 5.60 |
| 201809 | 438 | 4.51 |  | 3,799 | 5.69 |  | 3,716 | 5.57 |
| 201810 | 542 | 5.58 |  | 3,796 | 5.69 |  | 3,755 | 5.63 |
| 201811 | 534 | 5.50 |  | 3,762 | 5.64 |  | 3,717 | 5.57 |
| 201812 | 521 | 5.36 |  | 3,702 | 5.55 |  | 3,698 | 5.54 |
| 201901 | 535 | 5.51 |  | 3,620 | 5.42 |  | 3,687 | 5.52 |
| 201902 | 494 | 5.08 |  | 3,589 | 5.38 |  | 3,755 | 5.63 |
| 201903 | 496 | 5.10 |  | 3,647 | 5.46 |  | 3,688 | 5.53 |

| *Table A5. Summary Statistics* | | | |
| --- | --- | --- | --- |
|  | Pregnancy | No Pregnancy | SMD |
| Credit Score | 558 | 559 | 0.01 |
| Probability of poor credit | 0.66 | 0.67 | 0.03 |
| Total unpaid medical debt | $758 | $856 | 0.06 |
| Total unpaid non-medical debt | $460 | $510 | 0.06 |
| Age | 26.1 | 32.2 | 0.87 |
| 18-24 | 0.47 | 0.20 | -0.60 |
| 25-29 | 0.26 | 0.23 | -0.09 |
| 30-34 | 0.17 | 0.20 | 0.08 |
| 35-39 | 0.08 | 0.17 | 0.28 |
| 40-44 | 0.02 | 0.12 | 0.41 |
| 45-49 | 0.01 | 0.09 | 0.41 |
| Race/Ethnicity |  |  |  |
| Non-Hispanic White | 0.36 | 0.39 | 0.07 |
| Non-Hispanic Black | 0.51 | 0.48 | -0.05 |
| Hispanic | 0.10 | 0.09 | -0.04 |
| Other | 0.04 | 0.04 | 0.00 |
| Urban | 0.74 | 0.73 | -0.02 |
| N (Individuals x Period) | 301,227 | 2,069,281 |  |
| N (Individuals) | 9,717 | 66,751 |  |
| *Note:* All financial outcomes represent the average over the 31-month observation window. Demographic characteristics are defined at baseline (Month -1). | | | |

| *Table A6. Impact of pregnancy on credit score* | | | | | | |
| --- | --- | --- | --- | --- | --- | --- |
|  | (A) Overall | (B) White | (C) Black | (D) Hispanic | (E) Urban | (F) Rural |
| Pregnancy x Month -9 | 1.4 (0.2 to 2.6) p=0.02 | 1.7 (-0.21 to 3.6) p=0.08 | 1.12 (-0.63 to 2.88) p=0.21 | 1.72 (-2.23 to 5.66) p=0.39 | 1.83 (0.42 to 3.24) p=0.01 | 0.2 (-2.12 to 2.51) p=0.87 |
| Pregnancy x Month -8 | 1.07 (-0.05 to 2.19) p=0.06 | 0.98 (-0.79 to 2.76) p=0.28 | 1.2 (-0.45 to 2.85) p=0.15 | 1.05 (-2.7 to 4.81) p=0.58 | 1.07 (-0.25 to 2.39) p=0.11 | 1.13 (-1.03 to 3.29) p=0.3 |
| Pregnancy x Month -7 | 0.85 (-0.2 to 1.91) p=0.11 | 0.89 (-0.77 to 2.55) p=0.29 | 0.93 (-0.62 to 2.47) p=0.24 | 0.25 (-3.25 to 3.75) p=0.89 | 0.79 (-0.44 to 2.01) p=0.21 | 1.09 (-0.96 to 3.14) p=0.3 |
| Pregnancy x Month -6 | 1.2 (0.22 to 2.18) p=0.02 | 1.52 (-0.03 to 3.06) p=0.06 | 0.81 (-0.62 to 2.25) p=0.27 | 1.55 (-1.7 to 4.8) p=0.35 | 1.14 (0 to 2.27) p=0.05 | 1.4 (-0.53 to 3.32) p=0.16 |
| Pregnancy x Month -5 | 1.03 (0.14 to 1.92) p=0.02 | 0.79 (-0.59 to 2.17) p=0.26 | 0.54 (-0.76 to 1.84) p=0.42 | 3.47 (0.4 to 6.53) p=0.03 | 1 (-0.03 to 2.03) p=0.06 | 1.12 (-0.63 to 2.88) p=0.21 |
| Pregnancy x Month -4 | 1.19 (0.39 to 1.99) p=0.00 | 1.17 (-0.06 to 2.39) p=0.06 | 0.67 (-0.5 to 1.83) p=0.26 | 3.03 (0.19 to 5.86) p=0.04 | 1.18 (0.25 to 2.11) p=0.01 | 1.23 (-0.35 to 2.8) p=0.13 |
| Pregnancy x Month -3 | 0.51 (-0.18 to 1.2) p=0.15 | 0.62 (-0.45 to 1.7) p=0.26 | 0.23 (-0.77 to 1.24) p=0.65 | 1.35 (-1 to 3.69) p=0.26 | 0.42 (-0.38 to 1.23) p=0.3 | 0.77 (-0.57 to 2.1) p=0.26 |
| Pregnancy x Month -2 | -0.11 (-0.63 to 0.4) p=0.67 | 0.22 (-0.6 to 1.04) p=0.6 | -0.51 (-1.26 to 0.24) p=0.18 | 0.73 (-1.03 to 2.49) p=0.42 | -0.1 (-0.71 to 0.51) p=0.75 | -0.14 (-1.12 to 0.84) p=0.78 |
| Pregnancy x Month -1 | (omitted) | (omitted) | (omitted) | (omitted) | (omitted) | (omitted) |
| Pregnancy x Month 0 | -0.35 (-0.85 to 0.15) p=0.17 | -0.28 (-1.07 to 0.5) p=0.48 | -0.55 (-1.27 to 0.18) p=0.14 | 0.12 (-1.51 to 1.75) p=0.88 | -0.26 (-0.84 to 0.32) p=0.38 | -0.59 (-1.56 to 0.38) p=0.23 |
| Pregnancy x Month 1 | -0.66 (-1.33 to 0.01) p=0.06 | -0.15 (-1.22 to 0.91) p=0.78 | -0.95 (-1.93 to 0.03) p=0.06 | 0.04 (-2.13 to 2.2) p=0.97 | -0.42 (-1.2 to 0.37) p=0.3 | -1.34 (-2.65 to -0.04) p=0.04 |
| Pregnancy x Month 2 | -0.31 (-1.09 to 0.46) p=0.43 | 0.35 (-0.84 to 1.54) p=0.57 | -0.64 (-1.78 to 0.5) p=0.27 | -0.05 (-2.59 to 2.48) p=0.97 | -0.1 (-1.01 to 0.81) p=0.83 | -0.94 (-2.43 to 0.55) p=0.22 |
| Pregnancy x Month 3 | -0.49 (-1.36 to 0.38) p=0.27 | -0.43 (-1.77 to 0.9) p=0.53 | -0.66 (-1.96 to 0.64) p=0.32 | 0.54 (-2.28 to 3.36) p=0.71 | -0.25 (-1.27 to 0.77) p=0.64 | -1.22 (-2.88 to 0.45) p=0.15 |
| Pregnancy x Month 4 | -1.12 (-2.06 to -0.18) p=0.02 | -0.35 (-1.79 to 1.09) p=0.63 | -2.02 (-3.42 to -0.61) p=0.01 | 0.9 (-2.09 to 3.88) p=0.56 | -1 (-2.1 to 0.1) p=0.07 | -1.48 (-3.29 to 0.33) p=0.11 |
| Pregnancy x Month 5 | -1.4 (-2.4 to -0.4) p=0.01 | -0.24 (-1.8 to 1.31) p=0.76 | -2.52 (-3.99 to -1.05) p<0.01 | -0.27 (-3.58 to 3.04) p=0.87 | -1.14 (-2.31 to 0.03) p=0.06 | -2.18 (-4.11 to -0.24) p=0.03 |
| Pregnancy x Month 6 | -1.31 (-2.37 to -0.24) p=0.02 | 0.19 (-1.49 to 1.88) p=0.82 | -2.77 (-4.32 to -1.22) p<0.01 | -0.27 (-3.84 to 3.3) p=0.88 | -1.11 (-2.36 to 0.13) p=0.08 | -1.91 (-3.96 to 0.14) p=0.07 |
| Pregnancy x Month 7 | -0.82 (-1.93 to 0.28) p=0.15 | 0.75 (-0.99 to 2.5) p=0.4 | -2.36 (-3.97 to -0.75) p<0.01 | 0.44 (-3.26 to 4.15) p=0.82 | -0.57 (-1.86 to 0.72) p=0.39 | -1.6 (-3.75 to 0.55) p=0.14 |
| Pregnancy x Month 8 | -0.86 (-2 to 0.28) p=0.14 | 0.9 (-0.9 to 2.71) p=0.33 | -2.59 (-4.25 to -0.93) p<0.01 | 0.47 (-3.35 to 4.28) p=0.81 | -0.51 (-1.84 to 0.81) p=0.45 | -1.86 (-4.12 to 0.39) p=0.11 |
| Pregnancy x Month 9 | -0.59 (-1.77 to 0.59) p=0.33 | 1.7 (-0.16 to 3.56) p=0.07 | -2.79 (-4.5 to -1.09) p<0.01 | 1.72 (-2.22 to 5.66) p=0.39 | -0.15 (-1.52 to 1.22) p=0.83 | -1.92 (-4.22 to 0.38) p=0.1 |
| Pregnancy x Month 10 | -0.94 (-2.15 to 0.28) p=0.13 | 1.27 (-0.64 to 3.18) p=0.19 | -3.07 (-4.83 to -1.3) p<0.01 | 1.12 (-3 to 5.24) p=0.59 | -0.46 (-1.88 to 0.96) p=0.53 | -2.38 (-4.74 to -0.02) p=0.05 |
| Pregnancy x Month 11 | -1.66 (-2.9 to -0.41) p=0.01 | 0.88 (-1.08 to 2.85) p=0.38 | -3.96 (-5.76 to -2.15) p<0.01 | -0.17 (-4.34 to 4) p=0.94 | -1.44 (-2.9 to 0.01) p=0.05 | -2.34 (-4.76 to 0.09) p=0.06 |
| Pregnancy x Month 12 | -2.11 (-3.4 to -0.82) p<0.01 | 0.4 (-1.65 to 2.45) p=0.7 | -4.39 (-6.26 to -2.51) p<0.01 | -0.18 (-4.42 to 4.07) p=0.94 | -1.87 (-3.38 to -0.36) p=0.02 | -2.86 (-5.35 to -0.38) p=0.02 |
| Pregnancy x Month 13 | -1.52 (-2.83 to -0.21) p=0.02 | 1.18 (-0.9 to 3.26) p=0.27 | -4.14 (-6.03 to -2.24) p<0.01 | 1.68 (-2.59 to 5.94) p=0.44 | -1.27 (-2.8 to 0.26) p=0.11 | -2.33 (-4.84 to 0.18) p=0.07 |
| Pregnancy x Month 14 | -1.16 (-2.48 to 0.17) p=0.09 | 1.38 (-0.72 to 3.49) p=0.2 | -3.73 (-5.65 to -1.81) p<0.01 | 1.22 (-3.13 to 5.57) p=0.58 | -0.82 (-2.37 to 0.72) p=0.3 | -2.19 (-4.75 to 0.36) p=0.09 |
| Pregnancy x Month 15 | 0.12 (-1.23 to 1.47) p=0.87 | 1.91 (-0.25 to 4.07) p=0.08 | -1.91 (-3.86 to 0.04) p=0.06 | 3.16 (-1.24 to 7.56) p=0.16 | 0.36 (-1.22 to 1.93) p=0.66 | -0.66 (-3.29 to 1.97) p=0.62 |
| Pregnancy x Month 16 | 0.35 (-1.02 to 1.71) p=0.62 | 1.83 (-0.37 to 4.03) p=0.1 | -1.61 (-3.58 to 0.36) p=0.11 | 3.55 (-0.85 to 7.95) p=0.11 | 0.38 (-1.21 to 1.97) p=0.64 | 0.13 (-2.54 to 2.81) p=0.92 |
| Pregnancy x Month 17 | 0.46 (-0.92 to 1.85) p=0.51 | 1.42 (-0.81 to 3.66) p=0.21 | -1.22 (-3.22 to 0.78) p=0.23 | 3.39 (-1.16 to 7.95) p=0.14 | 0.63 (-0.98 to 2.24) p=0.44 | -0.18 (-2.89 to 2.52) p=0.89 |
| Pregnancy x Month 18 | 0.94 (-0.46 to 2.35) p=0.19 | 2.26 (-0.01 to 4.53) p=0.05 | -0.7 (-2.72 to 1.33) p=0.5 | 3.17 (-1.51 to 7.86) p=0.19 | 0.93 (-0.72 to 2.57) p=0.27 | 0.84 (-1.89 to 3.57) p=0.55 |
| Pregnancy x Month 19 | 1.34 (-0.09 to 2.76) p=0.07 | 2.25 (-0.06 to 4.57) p=0.06 | -0.29 (-2.34 to 1.76) p=0.78 | 4.75 (0.03 to 9.48) p=0.05 | 1.47 (-0.19 to 3.14) p=0.08 | 0.8 (-1.98 to 3.57) p=0.57 |
| Pregnancy x Month 20 | 2.04 (0.59 to 3.5) p=0.01 | 2.46 (0.11 to 4.81) p=0.04 | 0.65 (-1.44 to 2.75) p=0.54 | 6.77 (1.95 to 11.58) p=0.01 | 2.27 (0.58 to 3.96) p=0.01 | 1.2 (-1.65 to 4.04) p=0.41 |
| Pregnancy x Month 21 | 2.02 (0.53 to 3.52) p=0.01 | 2.04 (-0.37 to 4.45) p=0.1 | 0.74 (-1.4 to 2.87) p=0.5 | 6.64 (1.53 to 11.76) p=0.01 | 2.4 (0.66 to 4.15) p=0.01 | 0.72 (-2.2 to 3.64) p=0.63 |
| N (Individuals x Period) | 2,370,508 | 920,080 | 1,149,790 | 206,956 | 1,739,627 | 630,881 |
| N (Individuals) | 76,468 | 29,680 | 37,090 | 6,676 | 56,117 | 20,351 |
| Joint pre-trend test  p-value | 0.19 | 0.24 | 0.15 | <0.01 | 0.22 | 0.27 |

| *Table A7. Impact of pregnancy on unpaid medical debt* | | | | | | |
| --- | --- | --- | --- | --- | --- | --- |
|  | (A) Overall | (B) White | (C) Black | (D) Hispanic | (E) Urban | (F) Rural |
| Pregnancy x Month -9 | 12.33 (-11.1 to 35.76) p=0.3 | 27.29 (-15.62 to 70.21) p=0.21 | 6.7 (-24.84 to 38.23) p=0.68 | 1.23 (-67.97 to 70.44) p=0.97 | 12.53 (-13.51 to 38.57) p=0.35 | 13.97 (-37.71 to 65.66) p=0.6 |
| Pregnancy x Month -8 | 1.67 (-20.06 to 23.4) p=0.88 | 14.14 (-24.91 to 53.19) p=0.48 | -1.14 (-30.82 to 28.55) p=0.94 | -15.86 (-78.75 to 47.03) p=0.62 | -0.49 (-24.78 to 23.8) p=0.97 | 9.94 (-37.31 to 57.19) p=0.68 |
| Pregnancy x Month -7 | 0.09 (-20.23 to 20.41) p=0.99 | 6.36 (-29.34 to 42.06) p=0.73 | 0.1 (-28.1 to 28.29) p=1 | -17.63 (-76.97 to 41.72) p=0.56 | 0.74 (-22.03 to 23.51) p=0.95 | -0.01 (-43.9 to 43.88) p=1 |
| Pregnancy x Month -6 | -10.69 (-28.51 to 7.14) p=0.24 | -11.63 (-41.05 to 17.78) p=0.44 | -6.93 (-32.63 to 18.77) p=0.6 | -24.36 (-78.32 to 29.61) p=0.38 | -11.17 (-31.63 to 9.29) p=0.29 | -7.14 (-43.45 to 29.17) p=0.7 |
| Pregnancy x Month -5 | -11.22 (-26.91 to 4.46) p=0.16 | -11.23 (-36.74 to 14.28) p=0.39 | -10.52 (-33.32 to 12.29) p=0.37 | -16.6 (-65.77 to 32.57) p=0.51 | -7.42 (-25.55 to 10.72) p=0.42 | -20.79 (-52.02 to 10.45) p=0.19 |
| Pregnancy x Month -4 | -4.61 (-18.1 to 8.88) p=0.5 | -3.95 (-25.59 to 17.68) p=0.72 | -1.29 (-20.92 to 18.34) p=0.9 | -20.18 (-62.88 to 22.51) p=0.35 | -3.01 (-18.48 to 12.47) p=0.7 | -8.69 (-36.18 to 18.8) p=0.54 |
| Pregnancy x Month -3 | -5.53 (-16.37 to 5.3) p=0.32 | -6.57 (-23.28 to 10.15) p=0.44 | -4.47 (-20.23 to 11.3) p=0.58 | -23.56 (-60.91 to 13.79) p=0.22 | -6.15 (-18.8 to 6.51) p=0.34 | -3.23 (-24.19 to 17.73) p=0.76 |
| Pregnancy x Month -2 | -4.56 (-12.26 to 3.14) p=0.25 | -9.72 (-21.41 to 1.98) p=0.1 | -1.28 (-12.53 to 9.98) p=0.82 | -6.3 (-35.53 to 22.93) p=0.67 | -7.43 (-16.22 to 1.35) p=0.1 | 3.93 (-11.98 to 19.83) p=0.63 |
| Pregnancy x Month -1 | (omitted) | (omitted) | (omitted) | (omitted) | (omitted) | (omitted) |
| Pregnancy x Month 0 | 0.52 (-8.31 to 9.35) p=0.91 | -0.82 (-16.71 to 15.08) p=0.92 | 1.24 (-10.71 to 13.2) p=0.84 | 5.19 (-23.71 to 34.08) p=0.73 | -4.62 (-15.4 to 6.16) p=0.4 | 14.96 (0.5 to 29.43) p=0.04 |
| Pregnancy x Month 1 | 3.34 (-8.75 to 15.43) p=0.59 | -1.37 (-20.17 to 17.43) p=0.89 | 4.06 (-14.09 to 22.21) p=0.66 | 20.09 (-18.84 to 59.02) p=0.31 | -0.93 (-15.45 to 13.6) p=0.9 | 14.92 (-6.32 to 36.17) p=0.17 |
| Pregnancy x Month 2 | 3.33 (-11.01 to 17.68) p=0.65 | 1.99 (-21.78 to 25.76) p=0.87 | 3.84 (-16.95 to 24.62) p=0.72 | 6.24 (-38.3 to 50.79) p=0.78 | 2.39 (-14.47 to 19.25) p=0.78 | 5.26 (-21.98 to 32.5) p=0.71 |
| Pregnancy x Month 3 | 9.85 (-6.23 to 25.93) p=0.23 | 3.67 (-22.35 to 29.69) p=0.78 | 9.02 (-14.22 to 32.27) p=0.45 | 25.56 (-28.85 to 79.97) p=0.36 | 10.65 (-8.38 to 29.68) p=0.27 | 6.61 (-23.2 to 36.43) p=0.66 |
| Pregnancy x Month 4 | 13.47 (-4.37 to 31.31) p=0.14 | 1.24 (-27.48 to 29.96) p=0.93 | 16.87 (-9.03 to 42.77) p=0.2 | 23.44 (-36.88 to 83.76) p=0.45 | 15.64 (-5.2 to 36.47) p=0.14 | 6.31 (-28.23 to 40.86) p=0.72 |
| Pregnancy x Month 5 | 13.34 (-5.15 to 31.82) p=0.16 | -3.42 (-34.01 to 27.18) p=0.83 | 17.82 (-8.56 to 44.21) p=0.19 | 39.39 (-23.23 to 102) p=0.22 | 16.24 (-5.18 to 37.66) p=0.14 | 4.13 (-32.52 to 40.79) p=0.83 |
| Pregnancy x Month 6 | 15.3 (-3.91 to 34.51) p=0.12 | -2.91 (-35.06 to 29.24) p=0.86 | 23.5 (-4.01 to 51.01) p=0.09 | 26.99 (-35.24 to 89.22) p=0.4 | 20.62 (-1.73 to 42.97) p=0.07 | -1.82 (-39.5 to 35.86) p=0.93 |
| Pregnancy x Month 7 | 15.26 (-5.06 to 35.57) p=0.14 | -9.13 (-42.98 to 24.72) p=0.6 | 26.19 (-2.99 to 55.37) p=0.08 | 37.17 (-27.21 to 101.54) p=0.26 | 23.17 (-0.1 to 46.44) p=0.05 | -9.74 (-51.34 to 31.87) p=0.65 |
| Pregnancy x Month 8 | 21.6 (0.26 to 42.94) p=0.05 | -4.75 (-40.3 to 30.81) p=0.79 | 32.64 (1.83 to 63.45) p=0.04 | 42.88 (-23.18 to 108.95) p=0.2 | 25.54 (1.28 to 49.8) p=0.04 | 8.22 (-36.34 to 52.77) p=0.72 |
| Pregnancy x Month 9 | 28.98 (6.74 to 51.23) p=0.01 | -1.38 (-38.51 to 35.74) p=0.94 | 43.37 (11.15 to 75.59) p=0.01 | 47.08 (-20.26 to 114.41) p=0.17 | 28.48 (3.32 to 53.63) p=0.03 | 28.42 (-18.61 to 75.46) p=0.24 |
| Pregnancy x Month 10 | 28.08 (5.19 to 50.96) p=0.02 | -1.75 (-39.4 to 35.89) p=0.93 | 45.75 (12.59 to 78.92) p=0.01 | 33.04 (-39.61 to 105.69) p=0.37 | 21.64 (-4.1 to 47.38) p=0.1 | 44.16 (-4.82 to 93.15) p=0.08 |
| Pregnancy x Month 11 | 24.82 (1.37 to 48.27) p=0.04 | -1.74 (-40.27 to 36.79) p=0.93 | 40.95 (6.98 to 74.92) p=0.02 | 28.77 (-46.45 to 103.98) p=0.45 | 15.02 (-11.44 to 41.49) p=0.27 | 50.9 (1.11 to 100.69) p=0.05 |
| Pregnancy x Month 12 | 27.23 (3.2 to 51.25) p=0.03 | -1.31 (-40.93 to 38.31) p=0.95 | 40.64 (6.09 to 75.19) p=0.02 | 43.55 (-36.16 to 123.27) p=0.28 | 16.65 (-10.52 to 43.81) p=0.23 | 55.26 (4.51 to 106.01) p=0.03 |
| Pregnancy x Month 13 | 25.97 (1.52 to 50.42) p=0.04 | 5.19 (-35.67 to 46.06) p=0.8 | 33.13 (-1.52 to 67.77) p=0.06 | 48.89 (-34.51 to 132.3) p=0.25 | 18.16 (-9.37 to 45.7) p=0.2 | 45.81 (-6.29 to 97.9) p=0.09 |
| Pregnancy x Month 14 | 19.8 (-5.05 to 44.65) p=0.12 | -2.84 (-44.99 to 39.3) p=0.9 | 27.64 (-7.39 to 62.67) p=0.12 | 48.97 (-33.79 to 131.74) p=0.25 | 9.47 (-18.62 to 37.56) p=0.51 | 46.49 (-5.99 to 98.97) p=0.08 |
| Pregnancy x Month 15 | 26.74 (0.9 to 52.58) p=0.04 | 9.45 (-34.21 to 53.11) p=0.67 | 27.27 (-9.31 to 63.85) p=0.14 | 72.51 (-13.56 to 158.59) p=0.1 | 21.28 (-8.01 to 50.58) p=0.15 | 39.19 (-14.99 to 93.37) p=0.16 |
| Pregnancy x Month 16 | 30.98 (4.33 to 57.63) p=0.02 | 8.2 (-37.18 to 53.58) p=0.72 | 34.64 (-2.61 to 71.9) p=0.07 | 82.49 (-9.42 to 174.4) p=0.08 | 23.99 (-6.32 to 54.3) p=0.12 | 48.19 (-7.22 to 103.59) p=0.09 |
| Pregnancy x Month 17 | 30.02 (2.97 to 57.07) p=0.03 | 8.33 (-37.88 to 54.54) p=0.72 | 32.24 (-5.37 to 69.85) p=0.09 | 81.36 (-12.45 to 175.17) p=0.09 | 25.55 (-5.34 to 56.43) p=0.11 | 40.02 (-15.64 to 95.68) p=0.16 |
| Pregnancy x Month 18 | 27.42 (-0.26 to 55.1) p=0.05 | 5.59 (-41.34 to 52.52) p=0.82 | 28.86 (-9.8 to 67.52) p=0.14 | 81.76 (-15.34 to 178.87) p=0.1 | 24.68 (-7 to 56.35) p=0.13 | 32.12 (-24.55 to 88.78) p=0.27 |
| Pregnancy x Month 19 | 22.94 (-5.17 to 51.06) p=0.11 | -5.55 (-52.66 to 41.57) p=0.82 | 32.84 (-6.9 to 72.58) p=0.11 | 56.49 (-40.66 to 153.63) p=0.25 | 23.91 (-8.49 to 56.3) p=0.15 | 16.87 (-39.61 to 73.34) p=0.56 |
| Pregnancy x Month 20 | 14.07 (-14.78 to 42.91) p=0.34 | -2.63 (-51.41 to 46.14) p=0.92 | 26.07 (-14.61 to 66.75) p=0.21 | -0.08 (-98.46 to 98.29) p=1 | 13.92 (-19.12 to 46.97) p=0.41 | 11.95 (-46.87 to 70.77) p=0.69 |
| Pregnancy x Month 21 | 8.54 (-21.13 to 38.21) p=0.57 | -9.99 (-59.73 to 39.74) p=0.69 | 19.21 (-22.44 to 60.87) p=0.37 | 11.15 (-93.42 to 115.72) p=0.83 | 9.81 (-24.22 to 43.83) p=0.57 | 3.41 (-56.89 to 63.71) p=0.91 |
| N (Individuals x Period) | 2,370,508 | 920,080 | 1,149,790 | 206,956 | 1,739,627 | 630,881 |
| N (Individuals) | 76,468 | 29,680 | 37,090 | 6,676 | 56,117 | 20,351 |
| Joint pre-trend test  p-value | 0.53 | 0.11 | 0.23 | <0.01 | 0.57 | 0.49 |

| *Table A8. Impact of pregnancy on unpaid non-medical debt* | | | | | | | |
| --- | --- | --- | --- | --- | --- | --- | --- |
|  | (A) Overall | (B) White | (C) Black | (D) Hispanic | (E) Urban | (F) Rural |  |
| Pregnancy x Month -9 | 7.06 (-6.3 to 20.42) p=0.3 | 2.87 (-15.41 to 21.15) p=0.76 | 11.15 (-10.09 to 32.38) p=0.3 | 14.27 (-27.4 to 55.95) p=0.5 | 14.55 (-1.01 to 30.11) p=0.07 | -14.61 (-40.67 to 11.45) p=0.27 |  |
| Pregnancy x Month -8 | 7.54 (-4.96 to 20.03) p=0.24 | 2.81 (-14.46 to 20.08) p=0.75 | 10.92 (-8.81 to 30.64) p=0.28 | 18.27 (-21.24 to 57.79) p=0.37 | 15.8 (1.18 to 30.41) p=0.03 | -16.41 (-40.45 to 7.63) p=0.18 |  |
| Pregnancy x Month -7 | 5.63 (-5.78 to 17.04) p=0.33 | 11.35 (-4.62 to 27.32) p=0.16 | 2.76 (-15.11 to 20.62) p=0.76 | 11.26 (-25.22 to 47.73) p=0.55 | 11.69 (-1.66 to 25.05) p=0.09 | -11.9 (-33.83 to 10.02) p=0.29 |  |
| Pregnancy x Month -6 | 7.35 (-3.13 to 17.84) p=0.17 | 8.86 (-5.24 to 22.97) p=0.22 | 7.21 (-9.43 to 23.86) p=0.4 | 10.94 (-22.9 to 44.78) p=0.53 | 13.43 (1.12 to 25.75) p=0.03 | -10.05 (-29.95 to 9.85) p=0.32 |  |
| Pregnancy x Month -5 | 2.7 (-6.83 to 12.23) p=0.58 | 10.54 (-1.75 to 22.83) p=0.09 | -1.91 (-17.14 to 13.32) p=0.81 | 3.13 (-29.06 to 35.32) p=0.85 | 7.16 (-4.14 to 18.46) p=0.21 | -9.93 (-27.49 to 7.62) p=0.27 |  |
| Pregnancy x Month -4 | 4.1 (-4.33 to 12.54) p=0.34 | 9.01 (-1.34 to 19.35) p=0.09 | 2.42 (-11.29 to 16.13) p=0.73 | 0.88 (-27.49 to 29.24) p=0.95 | 8.02 (-2.06 to 18.09) p=0.12 | -7.03 (-22.17 to 8.12) p=0.36 |  |
| Pregnancy x Month -3 | -4.57 (-11.63 to 2.5) p=0.21 | 6.35 (-3.02 to 15.72) p=0.18 | -12.69 (-24.13 to -1.26) p=0.03 | 1.86 (-19.3 to 23.02) p=0.86 | -2.94 (-11.39 to 5.51) p=0.5 | -9.02 (-21.7 to 3.65) p=0.16 |  |
| Pregnancy x Month -2 | -2.26 (-7.42 to 2.89) p=0.39 | 0.21 (-7.11 to 7.54) p=0.96 | -5.11 (-13.36 to 3.15) p=0.23 | 6.56 (-7.82 to 20.93) p=0.37 | -1.49 (-7.68 to 4.7) p=0.64 | -4.47 (-13.57 to 4.62) p=0.34 |  |
| Pregnancy x Month -1 | (omitted) | (omitted) | (omitted) | (omitted) | (omitted) | (omitted) |  |
| Pregnancy x Month 0 | -0.39 (-6.61 to 5.84) p=0.9 | -4.93 (-14.09 to 4.23) p=0.29 | 3.33 (-6.2 to 12.85) p=0.49 | 0.97 (-18.25 to 20.18) p=0.92 | 2.78 (-4.46 to 10.03) p=0.45 | -9.36 (-21.5 to 2.79) p=0.13 |  |
| Pregnancy x Month 1 | 2.03 (-5.83 to 9.89) p=0.61 | -2.48 (-13.85 to 8.89) p=0.67 | 8.22 (-3.98 to 20.41) p=0.19 | -5.63 (-29.13 to 17.88) p=0.64 | 5.7 (-3.55 to 14.95) p=0.23 | -8.3 (-23.17 to 6.57) p=0.27 |  |
| Pregnancy x Month 2 | 6.73 (-2.46 to 15.91) p=0.15 | 0.03 (-12.54 to 12.6) p=1 | 14.84 (0.43 to 29.24) p=0.04 | 0.55 (-30.36 to 31.46) p=0.97 | 14.54 (3.89 to 25.18) p=0.01 | -15.56 (-33.72 to 2.6) p=0.09 |  |
| Pregnancy x Month 3 | 8.36 (-2.04 to 18.76) p=0.12 | -1.52 (-15.37 to 12.33) p=0.83 | 19.31 (2.82 to 35.79) p=0.02 | 2.79 (-31.27 to 36.85) p=0.87 | 17.13 (5.02 to 29.24) p=0.01 | -16.65 (-36.9 to 3.59) p=0.11 |  |
| Pregnancy x Month 4 | 13 (1.84 to 24.16) p=0.02 | 0.41 (-14.3 to 15.11) p=0.96 | 23.54 (5.96 to 41.12) p=0.01 | 22.88 (-14.42 to 60.18) p=0.23 | 22.27 (9.25 to 35.29) p<0.01 | -13.23 (-34.83 to 8.38) p=0.23 |  |
| Pregnancy x Month 5 | 12.8 (1.03 to 24.58) p=0.03 | -2.4 (-18.06 to 13.27) p=0.76 | 26.36 (7.69 to 45.03) p=0.01 | 15.11 (-22.2 to 52.41) p=0.43 | 20.55 (6.76 to 34.34) p<0.01 | -8.93 (-31.51 to 13.65) p=0.44 |  |
| Pregnancy x Month 6 | 13.37 (1.02 to 25.71) p=0.03 | -2.41 (-18.86 to 14.04) p=0.77 | 28.46 (8.86 to 48.06) p<0.01 | 20.27 (-18.18 to 58.73) p=0.3 | 21.85 (7.38 to 36.31) p<0.01 | -10.37 (-33.97 to 13.24) p=0.39 |  |
| Pregnancy x Month 7 | 14.95 (2.06 to 27.84) p=0.02 | -1.81 (-18.93 to 15.32) p=0.84 | 32.35 (11.79 to 52.91) p<0.01 | 13.92 (-25.67 to 53.51) p=0.49 | 24.91 (9.87 to 39.96) p<0.01 | -13.01 (-37.95 to 11.92) p=0.31 |  |
| Pregnancy x Month 8 | 19.05 (5.69 to 32.42) p=0.01 | -0.07 (-17.88 to 17.73) p=0.99 | 37.84 (16.49 to 59.18) p<0.01 | 16.71 (-23.86 to 57.29) p=0.42 | 29.89 (14.24 to 45.54) p<0.01 | -11.2 (-36.76 to 14.36) p=0.39 |  |
| Pregnancy x Month 9 | 20.55 (6.65 to 34.45) p<0.01 | 1.85 (-16.81 to 20.52) p=0.85 | 39.45 (17.3 to 61.61) p<0.01 | 22.35 (-20.59 to 65.3) p=0.31 | 31.01 (14.7 to 47.31) p<0.01 | -8.48 (-34.94 to 17.99) p=0.53 |  |
| Pregnancy x Month 10 | 23.74 (9.35 to 38.12) p<0.01 | 4.47 (-14.99 to 23.94) p=0.65 | 42.51 (19.6 to 65.42) p<0.01 | 23.87 (-20.09 to 67.82) p=0.29 | 35 (18.13 to 51.86) p<0.01 | -7.64 (-35.08 to 19.8) p=0.59 |  |
| Pregnancy x Month 11 | 27.98 (13.16 to 42.79) p<0.01 | 3.27 (-16.32 to 22.86) p=0.74 | 50.39 (26.68 to 74.11) p<0.01 | 25.08 (-21.28 to 71.44) p=0.29 | 41.32 (23.92 to 58.73) p<0.01 | -9.45 (-37.49 to 18.6) p=0.51 |  |
| Pregnancy x Month 12 | 25.28 (9.91 to 40.66) p<0.01 | -2.86 (-23.41 to 17.69) p=0.79 | 50.11 (25.73 to 74.49) p<0.01 | 27.52 (-22.95 to 77.98) p=0.29 | 39.32 (21.12 to 57.53) p<0.01 | -14.07 (-42.44 to 14.3) p=0.33 |  |
| Pregnancy x Month 13 | 26.61 (10.86 to 42.37) p<0.01 | 1.23 (-20.36 to 22.81) p=0.91 | 49.37 (24.5 to 74.24) p<0.01 | 31.18 (-19.31 to 81.67) p=0.23 | 39.59 (20.98 to 58.2) p<0.01 | -9.63 (-38.92 to 19.66) p=0.52 |  |
| Pregnancy x Month 14 | 28.65 (12.41 to 44.9) p<0.01 | -1.31 (-23.93 to 21.31) p=0.91 | 55.26 (29.82 to 80.71) p<0.01 | 30.17 (-22.35 to 82.68) p=0.26 | 40.29 (21.21 to 59.36) p<0.01 | -3.7 (-34.47 to 27.08) p=0.81 |  |
| Pregnancy x Month 15 | 25.21 (8.6 to 41.81) p<0.01 | -0.89 (-23.73 to 21.95) p=0.94 | 48.24 (22.18 to 74.29) p<0.01 | 26.85 (-27.63 to 81.32) p=0.33 | 36.32 (16.79 to 55.85) p<0.01 | -5.37 (-36.7 to 25.95) p=0.74 |  |
| Pregnancy x Month 16 | 26.91 (9.9 to 43.92) p<0.01 | -2.75 (-25.78 to 20.27) p=0.82 | 54.25 (27.59 to 80.91) p<0.01 | 23.48 (-34.39 to 81.35) p=0.43 | 37.95 (18.02 to 57.87) p<0.01 | -3.46 (-35.97 to 29.04) p=0.84 |  |
| Pregnancy x Month 17 | 26.18 (8.86 to 43.5) p<0.01 | 2.12 (-21.45 to 25.68) p=0.86 | 51.17 (24.13 to 78.21) p<0.01 | 11.38 (-48.3 to 71.07) p=0.71 | 38.11 (17.77 to 58.45) p<0.01 | -6.84 (-39.67 to 25.99) p=0.68 |  |
| Pregnancy x Month 18 | 24.25 (6.56 to 41.94) p=0.01 | 1.23 (-22.92 to 25.37) p=0.92 | 47.99 (20.42 to 75.56) p<0.01 | 9.27 (-51.25 to 69.79) p=0.76 | 38.28 (17.42 to 59.13) p<0.01 | -14.9 (-48.01 to 18.21) p=0.38 |  |
| Pregnancy x Month 19 | 22.02 (3.95 to 40.09) p=0.02 | 0.35 (-24.66 to 25.36) p=0.98 | 46.93 (18.78 to 75.08) p<0.01 | -4.61 (-64.31 to 55.09) p=0.88 | 37.72 (16.31 to 59.14) p<0.01 | -21.98 (-55.25 to 11.3) p=0.2 |  |
| Pregnancy x Month 20 | 20.99 (2.46 to 39.52) p=0.03 | -3.05 (-28.68 to 22.58) p=0.82 | 47.03 (18.32 to 75.74) p<0.01 | -0.84 (-64.71 to 63.02) p=0.98 | 38.08 (16.16 to 60) p<0.01 | -27.46 (-61.73 to 6.81) p=0.12 |  |
| Pregnancy x Month 21 | 18.87 (-0.17 to 37.92) p=0.05 | -5.19 (-31.52 to 21.14) p=0.7 | 44.44 (14.79 to 74.1) p<0.01 | -5.61 (-70.14 to 58.93) p=0.87 | 34.16 (11.66 to 56.65) p<0.01 | -24.26 (-59.66 to 11.14) p=0.18 |  |
| N (Individuals x Period) | 2,370,508 | 920,080 | 1,149,790 | 206,956 | 1,739,627 | 630,881 |  |
| N (Individuals) | 76,468 | 29,680 | 37,090 | 6,676 | 56,117 | 20,351 |  |
| Joint pre-trend test  p-value | 0.43 | 0.10 | 0.07 | <0.01 | 0.41 | 0.53 |  |

*Figure A1: Credit Score, by Race and Residence*

| Urban Black | Rural Black  |
| --- | --- |
| Urban White   | Rural White  |
|  |  |

*Figure A2: Medical Debt, by Race and Residence*

| Urban Black  | Rural Black  |
| --- | --- |
| Urban White   | Rural White  |
|  |  |

*Figure A3: Non-Medical Debt, by Race and Residence*

| Urban Black  | Rural Black  |
| --- | --- |
| Urban White   | Rural White  |
|  |  |

*Figure A4: Credit Score, Excluding the COVID-19 Pandemic Period*

| *Panel A: Overall*   | *Panel D: Hispanic*   |
| --- | --- |
| *Panel B: White*    *Panel C: Black*   | *Panel E: Urban*    *Panel F: Rural*   |

*Figure A5: Medical Debt, Excluding the COVID-19 Pandemic Period*

| *Panel A: Overall*   | *Panel D: Hispanic*   |
| --- | --- |
| *Panel B: White*   | *Panel E: Urban*   |
| *Panel C: Black*   | *Panel F: Rural*   |

*Figure A6: Non-Medical Debt, Excluding the COVID-19 Pandemic Period*

| *Panel A: Overall*   | *Panel D: Hispanic*   |
| --- | --- |
| *Panel B: White*   | *Panel E: Urban*   |
| *Panel C: Black*   | *Panel F: Rural*   |

*Figure A7: Credit Score among women aged 18–40*

| *Panel A: Overall*   | *Panel D: Hispanic*   |
| --- | --- |
| *Panel B: White*    *Panel C: Black*   | *Panel E: Urban*    *Panel F: Rural*   |

*Figure A8: Medical Debt among women aged 18–40*

| *Panel A: Overall*   | *Panel D: Hispanic*   |
| --- | --- |
| *Panel B: White*   | *Panel E: Urban*   |
| *Panel C: Black*   | *Panel F: Rural*   |

*Figure A9: Non-Medical Debt among women aged 18–40*

| *Panel A: Overall*   | *Panel D: Hispanic*   |
| --- | --- |
| *Panel B: White*   | *Panel E: Urban*   |
| *Panel C: Black*   | *Panel F: Rural*   |

*Figure A10: Credit Score, Complete Cases Without Imputation*

| *Panel A: Overall*   | *Panel D: Hispanic*   |
| --- | --- |
| *Panel B: White*    *Panel C: Black*   | *Panel E: Urban*    *Panel F: Rural*   |

*Figure A11: Medical Debt, Complete Cases Without Imputation*

| *Panel A: Overall*   | *Panel D: Hispanic*   |
| --- | --- |
| *Panel B: White*   | *Panel E: Urban*   |
| *Panel C: Black*   | *Panel F: Rural*   |

*Figure A12: Non-Medical Debt, Complete Cases Without Imputation*

| *Panel A: Overall*   | *Panel D: Hispanic*   |
| --- | --- |
| *Panel B: White*   | *Panel E: Urban*   |
| *Panel C: Black*   | *Panel F: Rural*   |

*Figure A13: Alternative Random Assignment of Control-Group Index Dates*

| Credit Score  | Medical Debt  |
| --- | --- |
| Non-Medical Debt  |  |
|  |  |
